# Combined healthy lifestyle score and risk of epigenetic aging: a discordant monozygotic twin study

**DOI:** 10.18632/aging.203022

**Published:** 2021-05-25

**Authors:** Hexiang Peng, Wenjing Gao, Weihua Cao, Jun Lv, Canqing Yu, Tao Wu, Shengfeng Wang, Zengchang Pang, Min Yu, Hua Wang, Xianping Wu, Liming Li

**Affiliations:** 1 Department of Epidemiology and Biostatistics, School of Public Health, Peking University Health Science Center, Beijing 100191, China; 2 Qingdao Center for Disease Control and Prevention, Qingdao 266033, China; 3 Zhejiang Center for Disease Control and Prevention, Hangzhou 310051, China; 4 Jiangsu Center for Disease Control and Prevention, Nanjing 210009, China; 5 Sichuan Center for Disease Control and Prevention, Chengdu 610041, China

**Keywords:** healthy lifestyle, DNA methylation age, epigenetic aging, twin, Chinese

## Abstract

We investigated whether lifestyle influences epigenetic aging in 143 monozygotic twin pairs discordant for the combined healthy lifestyle score. Twins were scored for four lifestyle factors as unhealthy or healthy; non-smoker, moderate drinker, adequate fruit and vegetable intake, and sufficient physical activity. The combined healthy lifestyle score was calculated for each participant by summing the binary score for each factor. Individual and co-twin analyses were used to assess the relationship between single or combined lifestyle scores, along with DNA methylation age acceleration (AA) calculated using Horvath’s and Li’s epigenetic clocks, focusing on AA and intrinsic epigenetic age acceleration (IEAA) measures. Compared with the twins that scored no or one healthy lifestyle point, those who scored four healthy lifestyle points had lower Li_IEAA with similar results observed in the co-twin analysis. No significant relationships were found in analyses based on Horvath’s clock, although the direction of correlations was consistent with that determined using Li’s clock. Smoking and drinking did not significantly affect DNA methylation AA; however, physical activity and intake of vegetables and fruits did, although the influence varied depending on the epigenetic clock. Our findings suggest that a healthy lifestyle may be an important way to delay aging and prevent age-related diseases.

## INTRODUCTION

DNA methylation (DNAm) is the most widely studied epigenetic phenomenon that plays an important role in human growth and aging processes [1]. Recently, an “epigenetic clock” based on specific age-related CpG sites was developed to calculate DNA methylation age (DNAm age), and this clock was considered a promising epigenetic aging biomarker [2]. Compared with the chronological age calculated by the calendar, DNAm age is more informative and representative as it reflects the actual aging of the human body more accurately [3]. The rates of epigenetic aging in people of the same chronological age vary due to the differences in genetic background, physical health condition, socioeconomic status, and other personal factors. When DNAm age is higher or lower than the chronological age, this indicates that the body is in an accelerated or a decelerated aging state, respectively. DNAm age acceleration (AA), the residual of linear regression or the difference between DNAm age and chronological age, is frequently used as an indicator of epigenetic aging. Higher AA increases the risk of mortality [4, 5] and susceptibility to various cancers [6–8], Alzheimer's disease [9], and other age-related diseases [10, 11].

Genetic factors and environmental stimuli affect DNAm age to varying degrees, and the influence of genetic factors is relatively fixed [2, 12]. Therefore, it is of profound significance to delve deeper into epigenetic aging from the perspective of modifiable lifestyle factors. According to Backett et al. [13], lifestyle may be viewed as a single type of behavior, or a set of behaviors, typical for an individual or a group. The Global Burden of Disease Study 2017 indicates that the important risk factors for non-communicable diseases include harmful alcohol use, smoking, inadequate fruit and vegetable intake, and insufficient physical activity [14]. Therefore, here, we mainly focused on four lifestyle factors; smoking, alcohol consumption, fruit and vegetable intake, and physical activity. Previous studies that investigated the associations between those lifestyle factors and epigenetic aging, yielded inconsistent results. For example, a systematic review and meta-analysis including 61 original studies that examined the relationships among environmental factors, lifestyle factors, and DNAm age showed no significant effect of smoking on DNAm age [15]. In contrast, an American cohort study found that smoking significantly accelerates DNAm age (β = −8.73, *P* < 0.001) [16]. Similar results were reported in the Melbourne Collaboration Cohort [17] and the UK National Health and Development Survey Cohort [18]. Therefore, the relationship between smoking and DNAm age needs to be further studied and clarified. As for drinking, a significant association was found between excessive alcohol consumption and DNAm AA in patients with alcohol dependence [19] and alcohol use disorders [20], whereas moderate drinking was shown to decelerate DNAm age [16]. Few studies have investigated the influence of physical activity and intake of vegetables and fruits on DNAm age. A UK study [21] reported no correlation between epigenetic age and the time spent being sedentary or physically active in older adults, whereas one meta-analysis [22] showed that locomotion-related DNAm may reverse the "epigenetic clock" as people age. The intake of carotenoids, fruits, fish, and poultry is significantly associated with DNAm age deceleration [17, 23]. Altered DNA methylation patterns are associated with the pathophysiology of aging and diseases, and dietary interventions may restore or prevent these processes [24].

However, little is known about the effects of combined lifestyle factors on DNAm age. To the best of our knowledge, no previous study has estimated the association between a combined healthy lifestyle and the risk of epigenetic aging. Additionally, the majority of studies were conducted in the general population, with little evidence derived from twin populations. It is unclear whether the effects of lifestyle factors on epigenetic aging would remain significant after controlling for genetic background. It is well-known that studies in twins have a natural advantage of identical genetic profiles and matching intrauterine and early-age environments. This is especially true for studies in monozygotic (MZ) twins, who receive 100% identical genetic material from their parents. In particular, the co-twin design conducted in discordant MZ twins is considered as a 1:1 matching case-control study, which is more powerful in matching potential confounders in genetic and familial environments [25]. Besides, a relatively small sample size is required when MZ twin pairs are the target population because of their high intra-class correlation [26]. Therefore, it is imperative to replicate or evaluate the associations between environmental stimuli and epigenetic aging in twin populations.

Therefore, we aimed to investigate whether lifestyle factors and combined healthy lifestyle score influence epigenetic aging in 143 MZ twin pairs with discordant healthy lifestyle score surveyed in the Chinese National Twin Registry (CNTR).

## RESULTS

Results of the descriptive analysis are presented in Tables 1–3. We calculated the healthy lifestyle score based on the definitions in Table 1, and a total of 143 MZ twin pairs with discordant healthy lifestyle scores (72.7% men and 27.3% women) were identified. In those twins, 65.7% were non-smokers, 55.2% had moderate alcohol consumption or never drank, 68.2% consumed ≥5 servings of vegetables and fruits per day (Table 1). The distribution of participants with different healthy lifestyle scores was as follows: 50 (17.5%) scored no or one points, 94 (32.9%) scored two points, 97 (33.9%) scored three points, and 45 (15.7%) scored four points (Table 2). The average score for all subjects was 2.45, and women scored higher (3.14) than men (2.19). Participants in the healthiest group who scored four points were likely to be younger, female, more educated, and to have lower weight, lower Engel coefficient, and lower risk of chronic diseases.

**Table 1:** Four factors of combined healthy lifestyle score in CNTR.

**Healthy lifestyle factor**	**Score**	**Interpretation of the score**	**Proportion (%)**
Smoking	0	Smoking: current smokers	34.3
	1	Nonsmoking: never smoked or quit smoking	65.7
Alcohol consumption	0	Daily consumption ≥25g/d for men or ≥15g/d for women	44.8
	1	Daily consumption <25g/d for men or <15g/d for women or non-drinker	55.2
Intake of vegetable and fruit	0	Daily consumed < 5 servings of fruits and vegetables per day	31.8
	1	Daily consumed ≥5 servings of fruits and vegetables per day	68.2
Physical activity	0	Total physical activity levels were in the bottom 50% of males or females respectively	44.4
	1	Total physical activity levels were in the top 50% of males or females respectively	55.6

**Table 2:** General characteristics of study participants according to the combined healthy lifestyle score.

**Characteristics**	**All**	**Mean score**	**0-1**	**2**	**3**	**4**	***P***
Number of participants	286	2.45	50	94	97	45	
Age, mean(year)							0.882
<50	162	2.46	52.0	57.4	58.8	55.6	
>=50	124	2.44	48.0	42.6	41.2	44.4	
Sex,%							<0.001
male	208	2.19	98.0	86.2	60.8	42.2	
female	78	3.14	2.0	13.8	39.2	57.8	
BMI,%							0.801
<24.0	130	2.50	60.0	55.3	51.5	53.3	
≥24.0	156	2.40	40.0	44.7	48.5	46.7	
Education,%							0.132
Primary schools and below	119	2.61	62.0	64.9	56.7	44.4	
Junior High school and above	167	2.34	38.0	35.1	43.3	55.6	
Marriage,%							0.460
Unmarried	24	2.58	6.0	9.6	6.2	13.3	
Married	262	2.44	94.0	90.4	93.8	86.7	
Engel coefficient,%							0.044
>0.5	102	2.33	28.0	29.8	22.7	22.2	
0.3~0.5	110	2.61	46.0	29.8	42.3	22.2	
<0.3	74	2.36	26.0	40.4	35.1	55.6	
history of chronic disease,%							0.286
No	190	2.52	62.0	60.6	71.1	73.3	
Yes	96	2.31	38.0	39.4	28.9	26.7	

**Table 3:** Descriptive statistics of epigenetic aging indicators according to the combined healthy lifestyle score.

**Characteristics**	**All**	**0-1**	**2**	**3**	**4**	***P***
Number of participants	286	50	94	97	45	
Chronological age (mean (SD))	48.9(10.6)	49.9(11.2)	49.4 (10.3)	48.6 (10.6)	47.4(10.6)	0.646
Horvath_mage (mean (SD))	53.6 (8.9)	54.1 (9.3)	54.0 (8.3)	53.6 (9.2)	52.3 (8.9)	0.720
Li_mage (mean (SD))	48.4 (10.6)	49.0 (10.7)	49.2 (10.4)	48.4(10.9)	46.2 (9.9)	0.446
Horvath_AA (mean (SD))	0.0 (3.7)	-0.3(3.9)	0.1 (3.0)	0.2 (4.3)	-0.2 (3.7)	0.860
Li_AA(mean (SD))	0.0 (4.3)	-0.3 (3.1)	0.4 (4.3)	0.2 (5.2)	-0.9 (3.0)	0.401
Horvath_IEAA (mean (SD))	0.0 (3.5)	0.2 (3.8)	0.1 (2.9)	0.1 (4.0)	-0.6 (3.5)	0.716
Li_IEAA (mean (SD))	0.0 (4.1)	-0.2 (3.1)	0.3 (4.1)	0.3 (4.8)	-1.0 (3.2)	0.268

Note: mage: DNA methylation age; AA:age acceleration; IEAA: intrinsic epigenetic age acceleration, Li and Horvath present two algorithms for calculating mage respectively.

**Figure 1:**
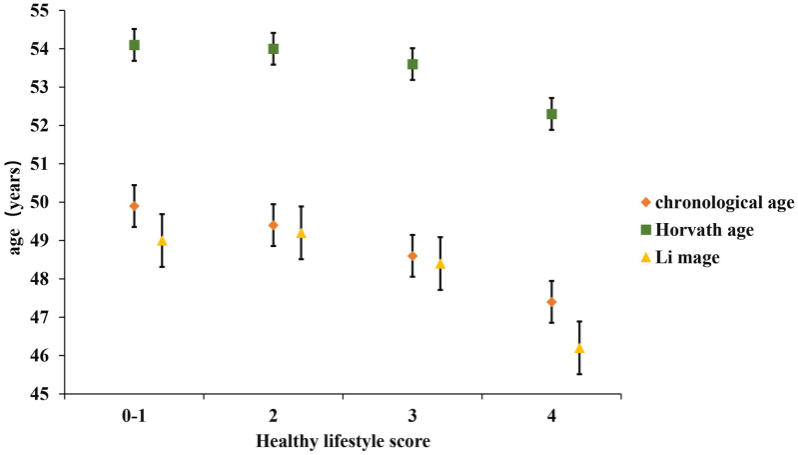
**Distribution of two kinds of predicted DNAm age and chronological age by healthy lifestyle score.** Data were shown as mean±standard error.

The participants were middle-aged with an average chronological age of 48.9 ± 10.6 years, and Li’s estimate of the DNAm age was very close to this value (48.4 ± 10.6 years), whereas Horvath’s predicted DNAm age had a larger deviation (Table 3). As expected, the chronological age was associated with DNAm age in both algorithms (Li_DNAm age: r = 0.91, error = 4.7, *P* = 1.3×10^−130^; Horvath_DNAm age: r = 0.91, error = 2.9, *P* = 1.3×10^−130^). Individuals with higher healthy lifestyle score were more likely to have lower predicted DNAm age (Figure 1).

We examined the association between each lifestyle factor (smoking, drinking, intake of vegetable and fruit, and physical activity) with each DNAm AA value calculated using Li’s and Horvath’s algorithms separately, adjusting for sex, chronological age, body mass index (BMI), education, Engel coefficient, and history of chronic disease as the mixed effects, and twin ID as the random effect. As shown in Supplementary Tables 1, 2 no association was found between smoking or drinking and any DNAm AA indicator. In contrast, a higher intake of vegetables and fruits was significantly associated with lower Li’s AA and IEAA. The top 20% of the participants had lower DNAm AA compared with participants in the bottom 20% in the mixed-effect model according to daily intake of vegetables and fruits (Li_AA: β = −1.70, *P* = 0.047, Li_IEAA: β = −1.66, *P* = 0.048). Similar results were observed in the co-twin analysis (Li_AA: β = −2.61, *P* = 0.024, Li_IEAA: β = −2.79, *P* = 0.013). As for physical activity, when the participants were divided into two groups by physical activity levels, the less active participants showed faster epigenetic aging (Li_AA: β = 1.84, *P* = 0.013, Li_IEAA: β = 1.86, *P* = 0.011). The results were also consistent in the paired analysis (Li_AA: β = 2.54, *P* = 0.007, Li_IEAA: β = 2.49, *P* = 0.007). Higher physical activity, especially for people with total physical activity levels between 60 and 80%, was significantly associated with Li_AA (β = −2.90, *P* = 0.020) and Li_IEAA (β = −2.52, *P* = 0.038) in co-twin analysis. However, we did not find significant associations between any single lifestyle factor and DNAm AA calculated using Horvath’s method.

**Figure 2:**
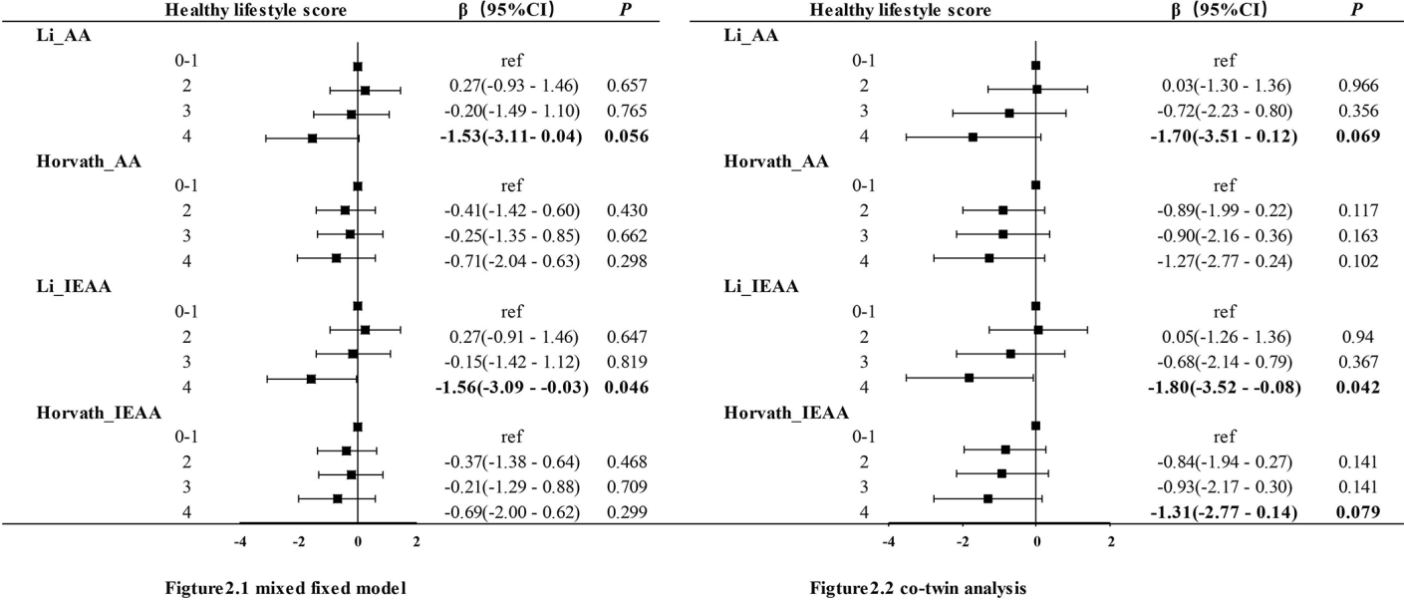
**Association between DNAm age acceleration and combined healthy lifestyle score.** Figure 2.1 presented the result generated by mixed effect model and the result of co-twin analysis were shown in Figure 2.2. AA:age acceleration; IEAA: intrinsic epigenetic age acceleration, Li and Horvath present two algorithms for calculating mage respectively.

In addition, when those single healthy lifestyle factors were calculated into a combined healthy lifestyle score, (Table 4 and Figure 2) higher combined healthy lifestyle score was significantly associated with slower epigenetic aging in both the mixed-effect model and co-twin analysis. In comparison with the twins that scored no or one point (unhealthiest group), the participants who scored four healthy lifestyle points had lower Li_IEAA (β = −1.56, *P* = 0.046) in the mixed model. In the co-twin analysis, the results were consistent with the previous analysis in the mixed-effect model (Li_IEAA: β = −1.80, *P* = 0.042). The same tendency was observed for the twins who scored four points (healthiest group) with lower Horvath_AA (β = −1.70, *P* = 0.069) and Horvath_IEAA (β = −1.31, *P* = 0.079), but the relationships were just short of the set level of statistical significance. Although the combined healthy lifestyle score was not associated with DNAm AA in the mixed-effect model when divided into two groups, the direction of the effect was negative, which was consistent with the results of the co-twin analysis. Compared with twins who scored 0–2 points, those who scored 3–4 points had lower Li_IEAA (β = −0.96, *P* = 0.049) and lower Li_AA (β = −0.93, *P* = 0.065) in the co-twin analysis. However, no association was observed between the combined healthy lifestyle score and Horvath’s DNAm AA measurement.

**Table 4:** Associations between DNAm age acceleration and combined lifestyle score.

**Healthy lifestyle score**	**Li_AA**			**Horvath_AA**			**Li_IEAA**			**Horvath_IEAA**	
	**β(95%CI)**	***P***		**β(95%CI)**	***P***		**β(95%CI)**	***P***		**β(95%CI)**	***P***
**mixed effect model**											
0-2	ref			ref			ref			ref	
3-4	-0.71(-1.59 - 0.17)	0.112		-0.03(-0.78 - 0.71)	0.928		-0.69(-1.55 - 0.17)	0.117		-0.04(-0.77 - 0.70)	0.922
**co-twin analysis**											
0-2	ref			ref			ref			ref	
3-4	**-0.93(-1.91 - 0.05)**	**0.065**		-0.22(-1.04 - 0.59)	0.594		**-0.96(-1.90 - -0.01)**	**0.049**		-0.33(-1.12 - 0.47)	0.423

Note: Table 4 shown the association between DNAm Age acceleration and dichotomy combined lifestyle score, and the results of multi-category combined lifestyle score were presented in Figure 2.

## DISCUSSION

In this study, we assessed the association between lifestyle factors and epigenetic aging in a cohort of twins from a population-based twin registry. A total of 143 MZ twin pairs with discordant healthy lifestyle score (208 men and 78 women) were included in our analysis. Our data provide new evidence of the association of the DNAm AA with smoking, drinking, intake of vegetables and fruits, physical activity, and the combined healthy lifestyle score calculated based on the individual lifestyle factors. We found that the intake of vegetables and fruits, physical activity, and the combined healthy lifestyle score were all independently and inversely associated with Li’s DNAm AA parameter. Only twins in the healthiest group (who scored one point on each of the four healthy lifestyle factors) were found marginally associated with Horvath’s IEAA. However, no association was observed between smoking, drinking, and any DNAm age measure. In agreement with previous studies [15, 27], the association between DNAm AA and phenotype or age-related diseases varied across different DNAm clocks. Distinct DNAm clocks might capture different aspects of aging when expressing the complicated relationship between environmental factors and age-related diseases [2, 28]. Drinking, smoking, and other components of the combined healthy lifestyle score may influence epigenetic aging through several different biological mechanisms, including modification of neurotrophic factors critical to epigenetic aging, oxidative stress, and modulation of various signaling pathways.

### Comparison of two epigenetic clocks

Significant associations between lifestyle factors and DNAm age parameters were mostly observed for Li’s epigenetic clock, but not for Horvath’s clock. The heterogeneity of the used epigenetic clocks might be an important factor leading to the difference in the results. Horvath’s epigenetic clock is the most widely used classical epigenetic clock in measuring biological aging. It is the first accurate multi-tissue biomarker of aging, which was developed from publicly available DNA methylation datasets to estimate the DNAm age for multiple tissues or organs [29]. As for Li’s clock, it is an accurate methylation age predictor specific for Chinese populations, but it is also validated to have high accuracy in twins from the CNTR [30]. However, it is worth noting that the correlation between the two epigenetic clocks was as high as 0.89 in the present study. Ethnicity, the number of CpG sites in the model, the statistical method of modeling epigenetic clocks, and the complex function of clock CpGs might lead to the difference of the results obtained by using different epigenetic clocks.

### Smoking, drinking, and DNAm age

In this study, smoking and drinking were not significantly associated with any DNAm age measures. Our results were consistent with the conclusions of other similar studies conducted in non-twin groups that also found no correlation between smoking and IEAA [23, 27, 31, 32], although two studies reported that smoking increases DNAm age [9, 17]. Smoking is known to affect DNA methylation patterns, but the evidence in favor of the significant relationship between DNAm age measures and smoking or other environmental exposures is inconsistent [15]. Similarly, the association between alcohol consumption and DNAm age was also inconsistent in different studies. There was no statistically significant difference in IEAA values between heavy drinkers and those who never drank in one study [32], whereas other studies observed faster DNAm AA in individuals with excessive alcohol consumption [19, 20].

### DNAm age and the intake of vegetables and fruits

At present, a limited number of studies focus on the influence of vegetables and fruits on epigenetic aging. A meta-analysis [15] including 7,493 participants from three studies [17, 23, 33] examined the association between diet and DNAm age, and showed that only the largest study of older women in the Women's Health Initiative [23] found a significant association between DNAm age and mean carotenoid levels, which was an index of vegetable intake. The Australian Cooperative Cohort Study that explored the relationship between multiple lifestyles and DNAm age found that fruit intake is associated with an increase in DNAm age, but no association of vegetable intake or physical activity with DNAm age was observed [17]. Fruits and vegetables are rich in folic acid and vitamins, and supplementation with folic acid and vitamin B_12_ can alter the DNA methylation profile [34]. Changes in DNA methylation patterns are associated with the pathophysiology of aging and diseases, and dietary interventions may restore or prevent these processes [24]. Additionally, socioeconomic factors might influence genomic DNA methylation in adults throughout the lifetime [35], as they influence dietary habits. Therefore, we adjusted for education and the Engel coefficient to control such confounders.

### Physical activity and DNAm age

This study suggested that physical activity might reduce epigenetic aging, which is consistent with the results of other studies that reported a negative association between DNAm age and parameters, such as step count [21] and grip strength [36, 37]. Another study conducted in discordant twins found that leisure-time physical activity is associated with slower epigenetic aging, whereas occupational physical activity is associated with faster aging [38, 39]. A bioinformatic meta-analysis reported that exercise-associated DNA methylation may “rewind” the epigenetic clock in the course of aging [22]. However, another meta-analysis [15] and a study by Zhao et al. [27] failed to replicate the association between physical activity and DNAm AA. These discrepancies might be explained by variable sample size, ethnicity, definition of physical activity, and other heterogeneous factors.

### Combined healthy lifestyle score and DNAm age

Compared with the unhealthiest participants, those who had a higher combined healthy lifestyle score tended to show slower epigenetic aging (DNAm age deceleration). Although no study has investigated the influence of the combined healthy lifestyle score on epigenetic aging so far, several previous studies found significant negative linear trends between the number of healthy lifestyle factors and the risks of cardiovascular disease and mortality [40, 41]. Consistent with our expectations and prior experience, DNAm age tended to decrease below the chronological age with the increase in the healthy lifestyle score. Due to the complex influence of multiple internal human body factors and external environmental factors on epigenetic parameters, the analysis of a single lifestyle factor is insufficient to reflect an authentic situation. Synergistic or antagonistic effects of different environmental factors may influence their combined action on the organism.

### Strengths and limitations

To the best of our knowledge, this is the first study to investigate the association between a combined healthy lifestyle score and epigenetic aging in discordant MZ twin pairs. We involved the combination of lifestyle factors instead of limiting our analysis to a single one. Discordant MZ twin design ensured an almost perfect matching of the genetic and early environmental factors in our analysis, which minimized the potential confounding effects. However, several limitations also need to be noted. First, our sample size was relatively small, though due to the discordant MZ design of this study, smaller sample size is required compared to a case-control study in the general population. Second, this was a cross-sectional study, which limited conclusions about causality relationships between lifestyle factors and DNAm age. Third, the BMI was not taken into consideration when calculating the combined healthy lifestyle score, because we regarded the BMI as an anthropometric indicator rather than a lifestyle factor.

## CONCLUSIONS

In conclusion, our study in discordant twins indicated that a combined lifestyle score was associated with epigenetic aging, and the healthiest participants (score 4) had slower DNAm AA than the unhealthiest group (score 0 or 1). In addition, the intake of vegetables and fruits, as well as physical activity, were inversely associated with epigenetic aging. Adherence to a healthy lifestyle may therefore slow down epigenetic aging, and this provides a new perspective in overcoming aging. Given the relatively small sample size and cross-sectional design of this study, further studies conducted in larger populations or using a prospective design are needed to address these issues in more detail.

## MATERIALS AND METHODS

### Study participants

The participants for this study were selected from the CNTR, the largest population-based twin registry in China, which had been previously described in more detail [42]. Data used in our analyses were collected in four provinces of China between 2011 and 2013 by trained investigators in community health service stations or the Centers for Disease Control and Prevention. Briefly, twins meeting the following criteria were included: (1) Preliminarily identified as MZ through a questionnaire with an accuracy of 0.87 [43]; (2) aged ≥18, with an available blood sample and questionnaire information; (3) with discordant combined healthy lifestyle score (quantitative details are provided below). Twins were excluded from our analysis if they were diagnosed with coronary heart disease, stroke, or cancer because these might modify their diet and health behaviors. Twins were excluded if their lifestyle information was missing. Finally, 143 twin pairs with discordant combined healthy lifestyle score were included in this study. All the twin pairs provided written informed consent, and the study protocol was approved by the Biomedical Ethics Committee of the Peking University (Number: IRB00001052-11029/13022).

### Measurement of lifestyle factors and other covariates

The lifestyle factors of interest were self-reported by twin participants using a uniform standardized questionnaire. Smoking was grouped into three categories (never smoker, current smoker, and former smoker). A similar classification was applied to drinking (never drinker, current drinker, and former drinker). In addition, current drinking was further classified using a continuous variable (alcohol consumption, g/day) and categorized into moderate (male, <25 g/day; female, <15 g/day) or excessive drinkers. For physical activity, we asked each participant about the extent and duration of each physical activity in different settings (working, commuting, domestic, and leisure-time). The total activity level was calculated by multiplying the metabolic equivalent tasks by the hours spent on each activity and then summing them up across different domains. For the intake of vegetables and fruits, investigators showed quantified pictures of different food items and asked them how many servings of fruits and vegetables they ate each day.

Potential covariates included chronological age, sex, BMI, education, Engel coefficient, and history of chronic disease. The BMI was calculated by dividing weight (kg) by height (m) squared and was classified into two groups: normal or underweight (BMI<24.0 kg/m^2^) and overweight or obese (BMI≥24 kg/m^2^). As a measure of a family's financial situation, the Engel coefficient was defined as the proportion of expenditure on household food out of total consumption, which was grouped into three categories (<0.3, 0.3–0.5, >0.5). Participants diagnosed with hypertension or diabetes were categorized as those with a history of chronic disease.

### Definition of the healthy lifestyle score

Four modifiable lifestyle factors of interest were included as follows: Smoking, drinking, physical activity, and intake of fruits and vegetables. For smoking and drinking, low-risk groups included current non-smokers, as well as never drinkers and moderate drinkers, respectively. The sex-specific median of the total physical activity level was used as a class boundary and the top 50% was classified as the low-risk group. For vegetables and fruits, those who consumed more than five servings (100 g/ serving) of fruits and vegetables per day were defined as the low-risk group. Finally, for each lifestyle factor, the low-risk group was defined as a healthy lifestyle and received a score of 1 (healthy), and otherwise obtained a score of 0 (unhealthy). The combined healthy lifestyle score was calculated for each participant by summing the binary score for each factor, ranging from 0 to 4, with 0 representing the unhealthiest group and 4 representing the healthiest group (Table 1). Due to the small number of participants with score 0, scores 0 and 1 were combined into the control group (score 0–1).

### DNA methylation quality control and processing

DNA extracted from whole blood was examined for methylation across the genome using the Illumina Human MethylationEPIC and Methylation450K BeadChips. DNA methylation levels were measured using the minfi package of R software in the form of β values, ranging from 0 to 1, where “0” represented completely unmethylated and “1” represented fully methylated. We excluded signal probes with a detection *P* value>0.01, cross-reactive probes, CpG sites associated with single-nucleotide polymorphisms, and CpG sites located on sex chromosomes. Samples with a missing rate >0.01 and those judged to originate from dizygotic twins according to 59 single-nucleotide polymorphisms on BeadChips were excluded. Finally, 143 MZ twin pairs with discordant healthy lifestyle score were included in further analyses.

### DNA methylation age and blood cell counts

Two algorithms proposed by Li et al. [30] and Horvath et al. [29] were applied to calculate DNAm age, also known as the “epigenetic age” or the “biological age”. Li’s algorithm was developed based on 239 age-related CpGs derived from whole blood samples in 989 Chinese and 160 Caucasian adults, providing accurate predictions for DNAm age in Chinese and Caucasians (R = 0.94–0.96, root mean square error = 3.8–4.3) [30]. Horvath’s algorithm (https://dnamage.genetics.ucla.edu/), built from multiple tissues and cell types, is based on methylation levels of 353 age-related CpGs from the Illumina 27k and 450k methylation arrays [29]. DNAm AA is regarded as the difference between DNAm age and chronological age, those with a DNAm AA higher than zero are described as exhibiting positive epigenetic age acceleration, whereas the reverse situation would be described as negative age acceleration [2]. To avoid the correlation with chronological age, DNAm AA was defined as the residuals of the linear regression model, in which DNAm age was defined as the outcome and chronological age as the independent variable. An important feature of blood that accompanies aging is the change in cell-type composition [2]. To reduce the potential effects of blood cell composition on AA, we generated intrinsic epigenetic age acceleration (IEAA) by additionally adjusting for blood cell counts based on the method proposed by Houseman et al. [44] in the regression procedure above. IEAA was considered to capture cell-intrinsic epigenetic aging independent of cell types.

### Statistical analysis

Data are presented as the mean ± standard deviation unless otherwise stated. A linear mixed model was used to examine the associations of four lifestyle factors (smoking, drinking, physical activity, intake of vegetables and fruits), and healthy lifestyle score with DNAm AA indicators generated by two epigenetic clocks separately (Horvath_AA, Horvath_IEAA, Li_AA and Li_IEAA). Adjusting factors included age, sex, education, Engel coefficient, history of chronic disease, BMI, and the clustering of twins was added as a random effect. The co-twin analysis was conducted in the discordant twin pairs using the fixed effect model, with the DNAm AA indicators as the outcome and lifestyle factors as the independent variables, respectively. A mixed-effect model was applied as an individual analysis and co-twin analysis was performed in twin pairs. The covariates were the same as those in the linear mixed model of co-twin analysis except for age and sex. The analysis of discordant MZ twins in biological and medical studies is a classic method for its unique advantage of subjects matched naturally by genetics, intrauterine environment, and early environmental factors. All the analyses were performed in R software 4.0.2. Effects were considered statistically significant if *P* < 0.05.

## Supplementary Material

Supplementary Tables
