# Supplementary material for: Combined healthy lifestyle score and risk of epigenetic aging: a discordant monozygotic twin study
Source: Aging (Albany NY). 2021 May 25;13(10):14039–52. doi: 10.18632/aging.203022 (PMC8202852; doi:10.18632/aging.203022)
Supplement: Supplementary Tables [file aging-13-203022-s001.pdf]

SUPPLEMENTARY TABLES

Supplementary Table 1. Associations between DNAm age acceleration and single lifestyle factors in mixed effect model.

Healthy lifestyle factor		Li_AA		Horvath_AA		Li_IEAA		Horvath_IEAA	
		β(95%CI)	*P*	β(95%CI)	*P*	β(95%CI)	*P*	β(95%CI)	*P*
**Smoking**	never	ref		ref		ref		ref	
	current	0.24(-1.05 - 1.54)	0.711	-0.46(-1.54 - 0.63)	0.504	0.17(-1.07 - 1.42)	0.781	-0.49(-1.53 - 0.55)	0.351
	former	0.44(-1.05 - 1.92)	0.561	-0.28(-1.52 - 0.97)	0.658	0.37(-1.10 - 1.85)	0.618	-0.32(-1.55 - 0.91)	0.604
**Drinking**	never or former	ref		ref		ref		ref	
	moderate	-1.05(-2.58 - 0.48)	0.178	0.05(-1.26 - 1.36)	0.941	-1.08(-2.58 - 0.43)	0.159	0.01(-1.28 - 1.31)	0.984
	excess	0.14(-0.96 - 1.24)	0.800	0.52(-0.42 - 1.46)	0.274	0.13(-0.95 - 1.22)	0.808	0.50(-0.43 - 1.43)	0.290
**Intake of vegetable and fruit**									
**two-category**	adequate	ref		ref		ref		ref	
	inadequate	0.71(-0.24 - 1.66)	0.143	0.17(-0.64 - 0.98)	0.674	0.67(-0.27 - 1.61)	0.163	0.16 (-0.65 - 0.96)	0.700
**multi-category**	<p20	ref		ref		ref		ref	
	P20-	**-2.07(-3.72 - -0.43)**	**0.014**	-0.74(-2.17 - 0.69)	0.306	**-1.97(-3.58 - -0.36)**	**0.017**	-0.78 (-2.18 - 0.62)	0.275
	P40-	-1.60(-3.41 - 0.20)	0.081	-0.71(-2.27 - 0.86)	0.375	-1.62(-3.41 - 0.16)	0.074	-0.75(-2.30 - 0.81)	0.342
	P60-	-0.66(-2.38- 1.06)	0.448	-0.68(-2.17 - 0.81)	0.366	-0.55(-2.25 - 1.14)	0.519	-0.70 (-2.18 - 0.78)	0.350
	P80-	**-1.70(-3.38 - -0.02)**	**0.047**	-0.98(-2.439 -0.48)	0.186	**-1.66(-3.31 - -0.01)**	**0.048**	-1.00(-2.44 - 0.43)	0.169
**Physical activity**									
**two-category**	active	ref		ref		ref		ref	
	inactive	**1.84(0.40 - 3.28)**	**0.013**	0.19(-0.62 - 0.99)	0.649	**1.86(0.42 - 3.29)**	**0.011**	-0.09(-1.35 - 1.16)	0.882
**multi-category**	<p20	ref		ref		ref		ref	
	P20-	0.52(-1.07 - 2.10)	0.520	-0.14(-1.51 - 1.23)	0.837	0.57(-1.00 - 2.12)	0.472	-0.09(-1.43 - 1.26)	0.901
	P40-	-0.55(-2.18 - 1.07)	0.501	0.02(-1.38 - 1.43)	0.974	-0.56(-2.17 - 1.05)	0.492	0.04(-1.36 - 1.43)	0.961
	P60-	-1.73(-3.81 - 0.35)	0.102	-1.15(-2.95 - 0.64)	0.206	-1.64(-3.71 - 0.43)	0.119	-1.08(-2.88 - 0.71)	0.234
	P80-	-1.42(-4.56 - 1.72)	0.371	-2.00(-4.71 - 0.71)	0.147	-1.27(-4.38 - 1.84)	0.420	-1.91(-4.61 - 0.79)	0.164

Supplementary Table 2. Associations between DNAm age acceleration and single lifestyle factors in co-twin analysis.

Healthy lifestyle factor		Li_AA		Horvath_AA		Li_IEAA		Horvath_IEAA	
		β(95%CI)	*P*	β(95%CI)	*P*	β(95%CI)	*P*	β(95%CI)	*P*
**Smoking**	never	ref		ref		ref		ref	
	current	0.36(-1.23 - 1.97)	0.653	-0.41(-1.78 - 0.95)	0.556	0.36(-1.14 - 1.86)	0.637	-0.26(-1.55 - 1.03)	0.691
	former	0.80(-1.03 - 2.64)	0.393	-0.51(-2.08 -1.06)	0.523	0.83(-0.95 - 2.62)	0.362	-0.57(-2.11 - 0.96)	0.467
**Drinking**	never or former	ref		ref		ref		ref	
	moderate	-0.87(-2.80 - 1.05)	0.375	-0.85(-2.47 - 0.78)	0.310	-0.86(-2.73 - 1.02)	0.372	-0.85(-2.47 - 0.78)	0.310
	heavy	0.65(-0.75 - 2.04)	0.367	0.22(-0.97 - 1.40)	0.721	0.64(-0.73 - 2.00)	0.362	0.22(-0.97 - 1.40)	0.721
**Intake of vegetable and fruit**									
**two-category**	adequate	ref		ref		ref		ref	
	inadequate	0.95(-0.19 - 2.09)	0.105	0.54(-0.41 - 1.48)	0.268	0.96(-0.16 - 2.08)	0.094	0.52(-0.42 - 1.45)	0.283
**multi-category**	<p20	ref		ref		ref		ref	
	P20-	**-2.43(-4.45 - -0.41)**	**0.020**	-0.14(-1.87 - 1.59)	0.874	**-2.47(-4.41 - -0.52)**	**0.014**	-0.63(-2.32 - 1.06)	0.468
	P40-	**-2.25(-4.65 - 0.16)**	**0.069**	-0.53(-2.60 - 1.53)	0.613	**-2.66(-4.97 - -0.34)**	**0.026**	-0.89(-2.90 - 1.13)	0.391
	P60-	-0.82(-3.00 - 1.37)	0.465	-0.07(-1.94 - 1.80)	0.944	-1.00(-3.12 - 1.12)	0.356	-0.60(-2.45 - 1.24)	0.522
	P80-	**-2.61(-4.84 - -0.38)**	**0.024**	-0.32(-2.23 - 1.60)	0.748	**-2.79(-4.95 - -0.63)**	**0.013**	-0.78(-2.67 - 1.10)	0.417
**Physical activity**									
**two-category**	active	ref		ref		ref		ref	
	inactive	**2.54(0.73 - 4.36)**	**0.007**	0.34(-1.24 - 1.92)	0.676	**2.49(0.71 - 4.28)**	**0.007**	0.60(-0.97 - 2.17)	0.452
**multi-category**	<p20	ref		ref		ref		ref	
	P20-	0.84(-1.14 - 2.82)	0.407	-0.42(-2.14 - 1.30)	0.634	1.08(-0.83 - 2.98)	0.269	-0.11(-1.78 - 1.56)	0.897
	P40-	-0.93(-2.85 - 0.99)	0.346	0.20(-1.47 - 1.87)	0.814	-0.68(-2.56 - 1.19)	0.477	-0.03(-1.67 - 1.62)	0.974
	P60-	**-2.90(-5.32 - 0.48)**	**0.020**	-1.44(-3.54 - 0.66)	0.181	**-2.52(-4.88 - -0.17)**	**0.038**	-1.61(-3.68 - 0.45)	0.129
	P80-	-2.78(-6.46 - 0.90)	0.142	-2.01(-5.20 - 1.18)	0.220	-2.17(-5.73 - 1.39)	0.235	-2.37(-5.49 - 0.75)	0.139
